# Statins and the Risk of Lung Cancer: A Meta-Analysis

**DOI:** 10.1371/journal.pone.0057349

**Published:** 2013-02-28

**Authors:** Min Tan, Xiaolian Song, Guoliang Zhang, Aimei Peng, Xuan Li, Ming Li, Yang Liu, Changhui Wang

**Affiliations:** Department of Respiratory Medicine, Shanghai Tenth People's Hospital, Tongji University, Shanghai, China; Università Vita-Salute San Raffaele, Italy

## Abstract

**Purpose:**

Several epidemiologic studies have evaluated the association between statins and lung cancer risk, whereas randomized controlled trials (RCTs) on cardiovascular outcomes provide relevant data as a secondary end point. We conducted a meta-analysis of all relevant studies to examine this association.

**Methods:**

A systematic literature search up to March 2012 was performed in PubMed database. Study-specific risk estimates were pooled using a random-effects model.

**Results:**

Nineteen studies (5 RCTs and 14 observational studies) involving 38,013 lung cancer cases contributed to the analysis. They were grouped on the basis of study design, and separate meta-analyses were conducted. There was no evidence of an association between statin use and risk of lung cancer either among RCTs (relative risk [RR] 0.91, 95% confidence interval [CI] 0.76–1.09), among cohort studies (RR 0.94, 95% CI 0.82–1.07), or among case-control studies (RR 0.82, 95% CI 0.57–1.16). Low evidence of publication bias was found. However, statistically significant heterogeneity was found among cohort studies and among case-control studies. After excluding the studies contributing most to the heterogeneity, summary estimates were essentially unchanged.

**Conclusion:**

The results of our meta-analysis suggest that there is no association between statin use and the risk of lung cancer.

## Introduction

Lung cancer is by far the most common cause of cancer mortality in the United States and throughout the world. According to the International Agency for Research on Cancer for 2008, about 1.6 million individuals were diagnosed with lung cancer and 1.4 million died as a result, which makes it the first-leading cause of cancer death in men and second in women globally [1]. In the United States, lung cancer is expected to account for 26% of all female cancer deaths and 29% of all male cancer deaths in 2012 [2].

Lung cancer stands out from other types of cancers because of our recognition of the major modifiable risk factor to the disease- exposure to tobacco smoke [3]. However, not all lung cancer cases are linked to cigarette smoking. Other risk factors include exposure to asbestos, haloethers, nickel, arsenic, and polycyclic aromatic hydrocarbons. Potential risk factors include genetic factors, dietary factors, and the presences of underlying benign forms of parenchymal lung diseases such as pulmonary fibrosis and chronic obstructive lung disease [4]–[5]. To date, no chemopreventive agent has been identified as an effective means to reduce the incidence of lung cancer.

Statins are inhibitors of 3-hydroxy-3-methyl glutaryl-coenzyme A reductase which is the rate-limiting enzyme in mevalonate synthesis. Statins are commonly used as cholesterol-lowering medications and have demonstrated the beneficial effects on cardiovascular morbidity and mortality [6]. As such, statins are some of the most widely prescribed drugs worldwide. Rodent studies suggested that statins may be carcinogenic [7]. In contrast, several preclinical studies indicate that these drugs may have cancer chemopreventive properties, through their interactions with essential cellular functions, such as cell proliferation and differentiation [8], [9]. Recently, meta-analysis of RCTs of statins for cardiovascular outcomes demonstrated no association between statin use and the risk of cancer [10]. However, the end-point of all cancers is not very sensitive and a negative finding does not suggest a lack of an effect at a particular site. Therefore, the effect of statins on the risk of lung cancer remains to be determined. To address this issue, we conducted a detailed meta-analysis of studies published in peer-reviewed literature.

## Materials and Methods

### Search Strategy

A systematic literature search up to March of 2012 was performed in PubMed database to identify relevant studies. Search terms included “HMG-CoA reductase inhibitor(s),” “statin(s)” combined with “cancer(s),” or “neoplasm(s)”. The search was limited to English language articles and those with human subjects. The title and abstract of studies identified in the search were scanned to exclude any clearly irrelevant studies. The full texts of the remaining articles were read to determine whether they contained information on the topic of interest. Furthermore, to find any additional published studies, a manual search was performed by checking all the references of retrieved articles. All searches were conducted independently by 2 authors (MT and XS). The results were compared, and any questions or discrepancies were resolved through iteration and consensus.

### Study Selection

To be eligible, studies had to fulfill the following 4 inclusion criteria: 1) RCTs or observational studies (case-control or cohort); 2) report results on statin use; 3) lung cancer incidence as the outcome of interest; and 4) reported the estimate of relative risk (RR) with their corresponding 95% confidence interval (CI) (or sufficient data to calculate of these effect measure). RCTs were considered eligible if they evaluated a statin therapy compared with placebo or no treatment, had no other intervention difference between the experimental and the control group. Studies reporting different measures of RR like risk ratio, rate ratio, hazard ratio (HR), and odds ratio (OR) were included in the meta-analysis. In practice, these measures of effect yield a similar estimate of RR, since the absolute risk of lung cancer is low.

### Data Extraction

Information from studies was extracted independently by 2 researchers (MT and XS), with disagreements resolved by consensus. The following data were collected: the first author’s last name, year of publication, country in which the study was performed, study design, years of follow-up or the study period, study participants age range, number of subjects and number of lung cancer cases, covariates controlled for in the analysis, and RR estimates with corresponding 95% CIs. If a study provided several risk estimates, the most completely adjusted estimate was extracted. Risk ratios and 95% CIs were calculated for each RCT by reconstructing contingency tables based on the number of participants randomly assigned and the number of participants with incident lung cancer (intention-to treat analysis). Differences in data extraction were resolved by consensus, referring back to the original article.

The quality of included RCTs was assessed based on Cochrane handbook [11], by recording seven items of bias risk: random sequence generation, allocation concealment, blinding of participants and personnel, blinding of outcome assessment, incomplete outcome data addressed, free of selective reporting, and free of other bias (follow-up ≥ 4 years). Each of the seven items is scored as ‘‘low risk,’’ ‘‘unclear risk,’’ or ‘‘high risk.” Meanwhile, the included cohort and case-control studies were assessed based on the 9-star Newcastle-Ottawa Scale for quality of non-randomized studies in meta-analyses [12].

### Statistical analysis

Studies were grouped on the basis of study design, and two separate meta-analyses were conducted: one meta-analysis of RCTs and a second meta-analysis of observational studies. This was done to examine consistency of results across varying study designs with different potential biases.

Study-specific risk estimates were extracted from each article, and log risk estimates were weighted by the inverse of their variances to obtain a pooled risk estimate. Studies were combined by using the DerSimonian and Laird random-effects model, which considers both within- and between-study variations [13].

Q and I^2^ statistics were used to examine whether the results of studies were homogeneous [14]. For the Q statistic, a *P* value < 0.10 was considered statistically significant for heterogeneity; for I^2^, a value >50% is considered a measure of severe heterogeneity. When statistical heterogeneity was detected, sensitivity analyses were performed. Publication bias was evaluated with Egger’s regression test in which *P* value less than 0.10 was considered representative of statistically significant publication bias [15]. All statistical analyses were performed with Stata software, version 10 (Stata Corp, College Station, Texas).

## Results

### Literature Search

**Figure 1:**
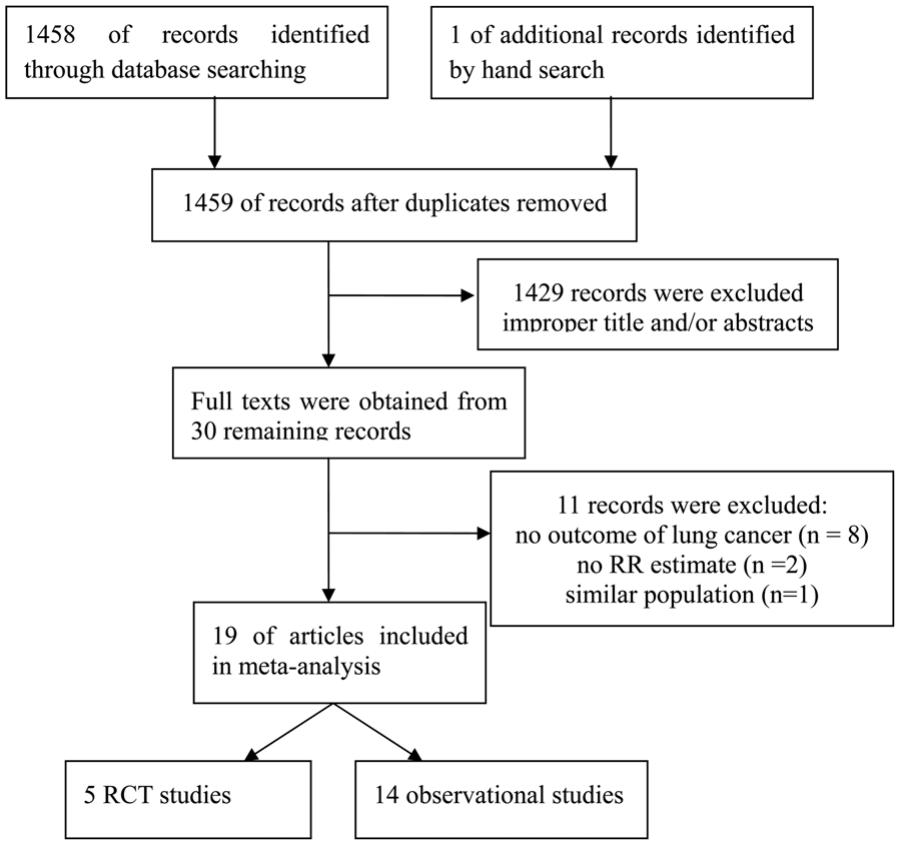
Flow diagram of study identification.

Our initial search strategy retrieved a total of 1459 citations. After the titles and abstracts were screened, 1429 articles were excluded because they were laboratory studies, review articles, or irrelevant to the current study. We identified 30 potentially relevant articles concerning statin use in relation to lung cancer risk. Eight publications were excluded because they investigated the association of statin with risk of total cancer and lung cancer was not among collected data [16]–[23]. Two articles were excluded because they did not provide RR estimate [24], [25] and one article was excluded because it reported on similar population [26]. Finally, 19 articles [27]–[45] concerning statin use and lung cancer risk (including 5 RCT studies and 14 observational studies) were included in this meta-analysis (Figure 1). We performed this meta-analysis in accordance with the guidelines of the Preferred Reporting Items for Systematic Reviews and Meta-analyses (PRISMA) statement (File 1) [46].

Four of five RCTs were placebo controlled, whereas one RCT [28] was a non-blinded trial comparing statin treatment with a usual care control group. All RCTs were multi-center trials and reported site-specific cancer outcomes (secondary end points) including lung cancer. Therefore, we were able to conduct a post hoc analysis of these trials and calculate risk ratios for lung cancer in an intention-to-treat analysis. Study designs, along with the RR estimates and 95% CIs, are listed in Table 1 for the RCTs and in Table 2 for the observational studies. Six observational studies [36]–[38], [40], [43], [44] were reported RR estimates of the association between long-term statin use and risk of lung cancer (Table 3).

**Table 1:** Randomized controlled trials included in the meta-analysis.

Study	Exposure	Duration (y)	Users	Non-users	Incident Lung Cancer		RR	95% CI
					Statins	Controls		
AFCAPS (27)	Lovastation	Mean,5.2	3,304	3,301	22	17	1.29	0.69–2.43
ALLHAT-LLT (28)	Pravastatin	Mean, 4.8	5,170	5,185	63	78	0.81	0.58–1.13
LIPS (29)	Fluvastatin	Median, 3.9	844	833	5	3	1.65	0.39–6.86
4S (30)	Simvastatin	Median, 10.4	2,221	2,223	25	31	0.81	0.48–1.36
WOSCOPS (31)	Pravastatin	Mean, 4.9	3,291	3,286	102	109	0.93	0.76–1.09

Abbreviations: RR, risk ratio; AFCAPS, Air Force/Texas Coronary Atherosclerosis Prevention Study; ALLHAT-LLT, Antihypertensive and Lipid-Lowering Treatment to Prevent Heart Attack Trial; LIPS, Lesol Intervention Prevention; 4S, Scandinavian Simvastatin Survival Study; HPS, Heart Protection Study; WOSCOPS, West of Scotland Coronary Prevention Study.

**Table 2:** Observational studies included in the meta-analysis.

Study	Year	Country	Design	Study period	Age, y	N. of participant	LC Cases	RR	95% CI	Adjustments*
Blais (32)	2000	Canada	C-C	1988–1994	≥65	5,962	70	0.94	0.43–2.05	1–6
Kaye (33)	2004	UK	C-C	1990–2002	50–89	18,088	259	0.9	0.6–1.3	1, 2, 7–9
Graaf (34)	2004	Netherlands	C-C	1985–1998	NR	20,105	445	0.89	0.56–1.42	1–5, 10–16
Friis (35)	2005	Denmark	Co	1989–2002	30–80	334,754	3,399	0.92	0.72–1.16	1, 2, 15, 16, 17
Coogan (36)	2007	US	C-C	1991–2005	40–79	8,813	464	0.7	0.4–1.1	1, 2, 8, 9, 15, 18, 19–22
Setoguchi (37)	2007	US	Co	1994–2003	>65	31,723	216	1.11	0.77–1.60	1, 2, 3, 7, 10, 11, 15, 19, 23–36
Khurana (38)	2007	US	C-C	1998–2004	18–100	483,733	7,280	0.55	0.52–0.59	1, 2, 8, 9, 10, 18, 19
Farwell (39)	2008	US	Co	1997–2005	66.5	62,842	867	0.70	0.60–0.81	1, 9, 10, 15, 33, 37–49
Friedman (40)	2008	US	Co	1994–2003	>20	361,859	1042	1.09†	0.96–1.23	50
Haukka (41)	2009	Finland	Co	1996–2005	60.0	944,962	5129	0.81	0.77–0.86	1, 2, 5
Hippisley (42)	2010	UK	Co	2002–2008	30–84	2,121,786	6001	1.03†	0.94–1.21	1, 8, 9, 51–54
Vinogradova (43)	2011	UK	C-C	1998–2008	30–100	450379	10,163	1.07	0.99–1.16	1, 2, 8, 9, 10, 15, 25, 39, 40, 49, 55
Jacobs (44)	2011	US	Co	1997–2007	>60	133,255	1,926	1.04†	0.95–1.14	1, 2, 8–10, 16, 19, 20, 39, 40, 56, 57
Cheng (45)	2012	Taiwan	C-C	2005–2008	>50	1485	297	0.82	0.58–1.15	1, 2, 4, 10, 15, 16, 58

Abbreviations: LC, lung cancer; RR, relative risk; C-C, case control; Co, cohort; NR, not reported.

* 1, age; 2, sex; 3, comorbidity score;4, other lipid-lowering therapy; 5, duration of follow-up; 6, history of neoplasia; 7, number of physician visits; 8, body mass index; 9, smoking status; 10, diabetes; 11, prior hospitalizations, 12, use of diuretics; 13, use of angiotensin-converting enzyme inhibitor; 14, use of calcium channel blockers; 15, use of nonsteroidal anti-inflammatory drugs; 16, hormone replacement therapy; 17, use of cardiovascular drugs; 18, alcohol use; 19, race; 20, education; 21, study center; 22, interview year; 23, inflammatory bowel disease; 24, benign mammary dysplasia; 25, arthritis; 26, use of gastroprotective drugs; 27, estrogen use; 28, obesity; 29; tobacco abuse; 30, mammography; 31, gynecologic examination; 32, Papanicolaou smear; 33, colonoscopy; 34, stool occult blood; 35, distinct generic medicines taken; 36, prior nursing home stay; 37, weight; 38, thyroid disease; 39, hypertension; 40 cardiovascular disease; 41, renal failure;42, chest pain; 43, mental illness; 44, alcoholism; 45, lung disease; 46, gastrointestinal disease; 47, prostate disease; 48, total cholesterol; 49, aspirin use ; 50,calendar year; 51,Townsend score, 52, any other cancer; 53,corticosteroids; 54, asthma; 55, Cox2-inhibitors; 56, physical activity; 57, history of elevated cholesterol; 58, tuberculosis.

† The risk estimate was calculated by post hoc analysis.

**Table 3:** Studies evaluating the association between long-term statin use and risk of lung cancer.

Study	LC cases	RR	95% CI	Definition of ‘‘long-term’’ statin use
Coogan (36)	10	0.9	0.4 to 2.1	>5 years
Setoguchi (37)	80	1.02	0.59 to 1.74	≥3 years
Khurana (38)	269	0.23	0.20 to 0.26	>4 years
Friedman (40)	119 (men)	1.06	0.88 to 1.28	>5 year
Friedman (40)	78 (women)	1.17	0.93 to 1.46	>5 year
Vinogradova (43)	558	1.18	1.05 to 1.34	≥49 months
Jacobs (44)	340	1.08	0.93 to 1.25	≥5 year

Abbreviations: LC, lung cancer; RR, relative risk. CI, confidence intervals.

Table 4 illustrates our opinion about each item of bias risk for included RCTs, most of the items were at ‘‘low risk’’ based on Cochrane handbook. Table 5 summarizes the quality scores of cohort studies and case-control studies based on the Newcastle-Ottawa Scale. Most of the observational studies score 5 or more, suggesting a reasonable good quality of the cohort and case-control studies.

**Table 4:** Methodological quality of included randomized controlled trials.*

Study	Random sequence generation	Allocation concealment	Blinding of participant and personal	Blinding of outcome assessment	Incomplete outcome data addressed	Free of selective reporting	Free of other bias†
AFCAPS (27)	Yes	Yes	Yes	Yes	Yes	Yes	Yes
ALLHAT-LLT (28)	Yes	Yes	Unclear	Yes	Yes	Yes	Yes
LIPS (29)	Yes	Yes	Yes	Yes	Yes	Yes	No
4S (30)	Yes	Unclear	Yes	Yes	Yes	Unclear	Yes
WOSCOPS (31)	Yes	Unclear	Yes	Yes	Yes	Yes	Yes

Abbreviations: AFCAPS, Air Force/Texas Coronary Atherosclerosis Prevention Study; ALLHAT-LLT, Antihypertensive and Lipid-Lowering Treatment to Prevent Heart Attack Trial; LIPS, Lesol Intervention Prevention; 4S, Scandinavian Simvastatin Survival Study; HPS, Heart Protection Study; WOSCOPS, West of Scotland Coronary Prevention Study.

* Yes, low risk of bias; Unclear, unclear risk of bias; No, high risk of bias.

† Follow-up ≥4 years.

**Table 5:** Methodological quality of included cohort studies and case–control studies based on the Newcastle–Ottawa Scale.

Cohort studies	Selection				Comparability	Outcome			Total score
	Representativeness of the exposed cohort	Selection of the non-exposed cohort	Ascertainment of exposure	Outcome of interest was not present at start of study	Control for important factor or additional factor	Assessment of outcome	Follow-up long enough for outcomes to occur †	Adequacy of follow-up of cohort	
Friis (35)	*	*		*	*	*		*	6
Setoguchi (37)	*	*	*	*	*	*		*	7
Farwell (39)	*	*	*	*	**	*	*	*	9
Friedman (40)	*	*		*		*	*	*	6
Haukka (41)	*	*		*	*	*	*	*	7
Hippisley (42)	*	*		*	**	*	*	*	8
Jacobs (44)	*	*		*	**	*	*	*	8

Case-control studies	Selection				Comparability	Exposure			Total score
	Adequate definition of cases	Representativenessof cases	Selection of controls	Definition of controls	Control for important factor or additional factor	Ascertainment of exposure	Same method of ascertainment for cases and controls	Non-response rate	
Blais (32)		*	*	*	*		*		5
Kaye (33)	*	*		*	**		*		6
Graaf (34)		*		*	*		*		4
Coogan (36)		*		*	**		*		5
Khurana (38)		*		*	**		*		5
Vinogradova (43)		*	*	*	**		*		6
Cheng (45)		*		*	*		*		4

† Follow-up ≥4 year.

### Meta-analysis of RCTs

**Figure 2:**
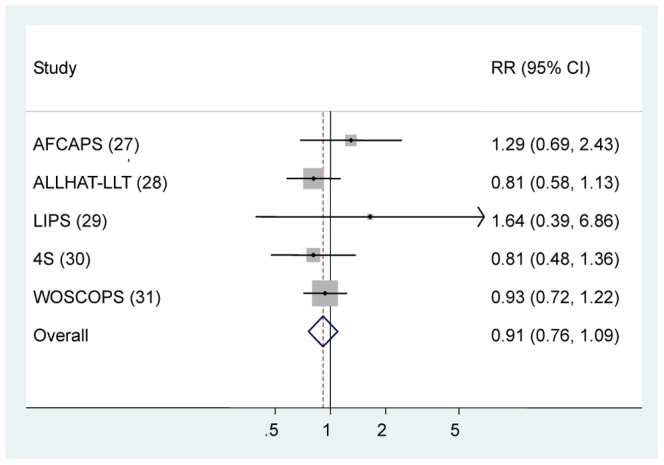
In RCT studies, risk estimates of lung cancer associated with statin use. Squares indicate study-specific risk estimates (size of the square reflects the study-specific statistical weight, i.e., the inverse of the variance); horizontal lines indicate 95% confidence intervals (CIs); diamonds indicate summary risk estimate with its corresponding 95% confidence interval. Abbreviations: RR, risk ratio; AFCAPS, Air Force/Texas Coronary Atherosclerosis Prevention Study; ALLHAT-LLT, Antihypertensive and Lipid-Lowering Treatment to Prevent Heart Attack Trial; LIPS, Lesol Intervention Prevention; 4S, Scandinavian Simvastatin Survival Study; HPS, Heart Protection Study; WOSCOPS, West of Scotland Coronary Prevention Study.

Five RCTs contributed to the analysis [27]–[31]. A total 29,658 individuals participated in these trials: 14,830 in treatment groups and 14,828 in control groups (Table 1). The participants had a mean follow-up of approximately 5.8 years. The overall rate of lung cancer was 1.46% in the statin group (217 incident cases) and 1.61% in the control group (238 incident cases). Figure 2 graphs the RR estimates and 95% CI from the individual trials and the pooled results. Statin use was not found to be associated with the risk of lung cancer (RR 0.91, 95% CI 0.76–1.09). The Cochran’s Q test resulted in a *P*  =  0.63 (Q  =  2.57), and the corresponding quantity I^2^ was 0%, both indicating that study results were homogeneous. The *P* value for the Egger test was *P*  =  0.30, suggesting a low probability of publication bias.

When the analysis was restricted to trials that evaluated statin therapy compared with placebo [27], [29]–[31], the results did not substantially change (RR 0.96, 95% CI 0.77–1.20). Similarly, after stratifying the data in two subgroups (lipophilic *v* lipophobic statins), we did not find any statistically significant association between lipophilic or lipophobic statins and lung cancer risk (Table 6).

**Table 6:** Meta-analysis results.

Study type	References	RR	95% CI	Heterogeneity test		
				*Q*	*P*	*I^2^* (%) †
RCTs	27–31	0.91	0.76 to 1.09	2.57	0.633	0
Placebo-controlled RCTs	27,29–31	0.96	0.77 to 1.20	1.86	0.601	0
RCTs of lipophilic statins	27,29,30	1.02	0.69 to 1.50	1.74	0.419	0
RCTs of lipophobic statins	28,31	0.88	0.72 to 1.09	0.44	0.509	0
Observational studies	32–45	0.88	0.75 to 1.04	267.72	<0.001	95.1
Cohort studies	35,37,39,40–42,44	0.94	0.82 to 1.07	49.09	<0.001	87.8
Case-control studies	32–34,36,38,43,45	0.82	0.57 to 1.16	169.01	<0.001	96.4
Long-term statin use	36–38, 40, 43, 44	0.81	0.42 to 1.56	416.93	<0.001	98.8
Adjust for smoking						
No	32,34,35,37,40,41,45	0.93	0.80 to 1.08	20.87	0.002	71.3
yes	33,36,38,39,42–44	0.84	0.64 to 1.11	237.68	<0.001	97.5

Abbreviations: RR, relative risk; CI, confidence intervals; RCT, randomized controlled trial.

† *I^2^* is interpreted as the proportion of total variation across studies that are due to heterogeneity rather than chance.

### Meta-Analysis of Observational Studies

The 14 relevant studies were published between 2000 and 2012 (Table 2) including 7 cohort studies [35], [37], [39], [40]–[42] and 7 case-control studies [32]–[34], [36], [38], [43], [45]. A total of 4,979,746 participants, including 37,558 lung cancer cases were involved in these studies and followed for 4–15 years. All studies evaluated exposure to statins and the risk of lung cancer except for one study [36] that examined the use of all cholesterol-lowering drugs. Five studies reported RR [32], [33], [35], [41], [44], 5 reported OR [34], [36], [38], [43], [45], and 4 reported HR [37], [39], [40], [42]. Most studies provided risk estimates that were adjusted for age (12 studies), sex (10 studies), smoking (7 studies), use of nonsteroidal anti-inflammatory drugs (7 studies), and diabetes (7 studies); fewer were adjusted for body mass index (6 studies), and alcohol use (2 studies) (Table 2).

**Figure 3:**
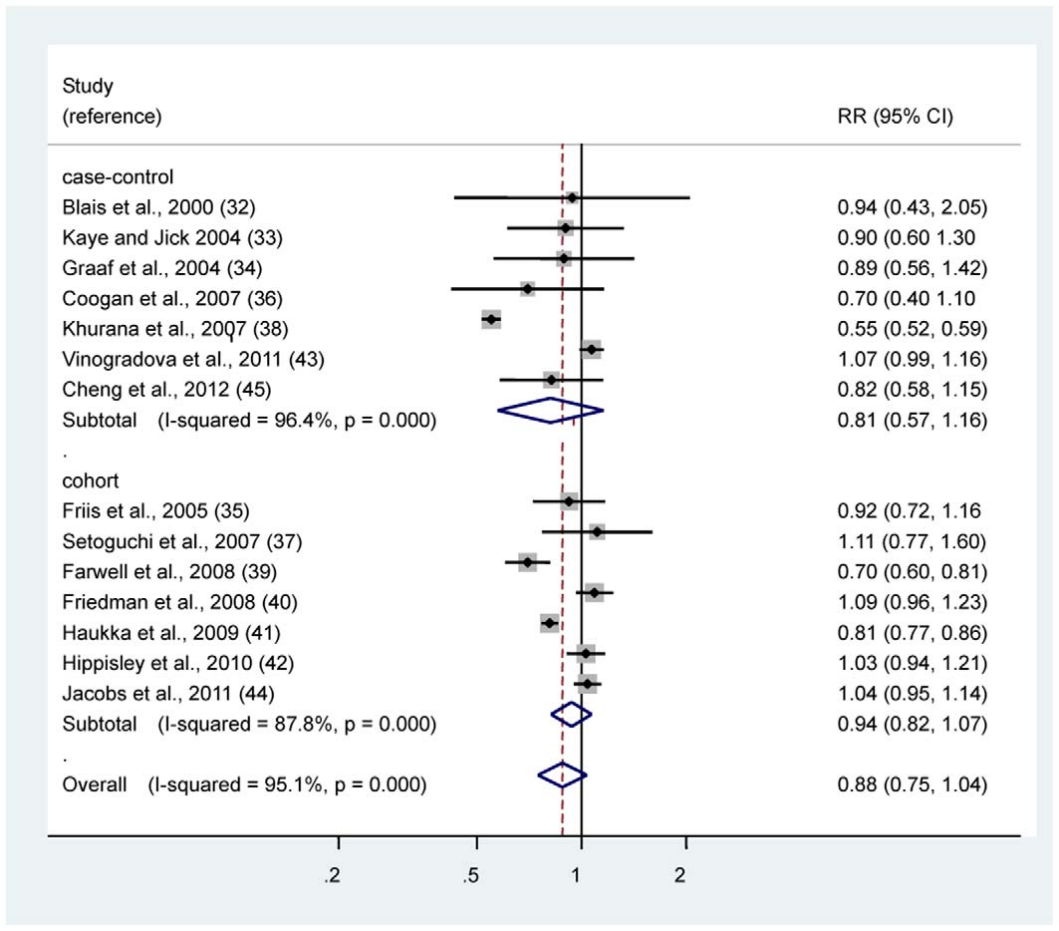
In observational studies, risk estimates of lung cancer associated with statin use. Squares indicate study-specific risk estimates (size of the square reflects the study-specific statistical weight, i.e., the inverse of the variance); horizontal lines indicate 95% confidence intervals (CIs); diamonds indicate summary risk estimate with its corresponding 95% confidence interval.

The multivariable-adjusted RRs of lung cancer for statin use in individual observational studies and summary estimates are shown in Figure 3. The overall RR of lung cancer for statin use was 0.88 (95% CI 0.75–1.04) for observational studies combined. There was statistically significant heterogeneity among studies (*P* < 0.001; I^2^  =  95.1%). The Egger test showed no evidence of publication bias (*P*  =  0.43).

To examine consistency across varying study designs with different potential biases, we stratified data into subgroups on the basis of study design. No significant association between statins and risk of lung cancer among case-control studies (RR 0.81, 95% CI 0.57–1.16) and cohort studies (RR 0.94, 95% CI 0.82–1.07). Significant heterogeneity was also observed among case-control studies (*P* < 0.001; I^2^  = 96.4%) and cohort studies (*P* < 0.001; I^2^  = 87.8%). By using a stepwise process, we determined that most of the heterogeneity was accounted for one study by Khurana et al. [38] in case-control studies. When this studies were excluded, the summary estimate was essentially unchanged (RR 0.99, 95% CI 0.87–1.11), but a concomitant shift in heterogeneity was measured by the Q-test (from *P* < 0.001 to *P*  =  0.335). And we also found that most of the heterogeneity was accounted for two studies by Farwell et al. [39] and by Haukka et al. [41] in cohort studies. When these two studies were excluded, the summary estimate was essentially unchanged (RR 1.04, 95% CI 0.98–1.11), but a concomitant shift in heterogeneity was measured by the Q-test (from *P* < 0.001 to *P*  =  0.790).

Further, six studies [36]–[38], [40], [43], [44] reported RR estimates of the association between long-term statin use and the risk of lung cancer (Table 3). Based on the results from these studies, the calculated combined RR for lung cancer in long-term statin use was found to be 0.81 (95 % CI 0.42–1.56) (Table 6). Stratified analysis by adjustment for smoking did not show any statistically significant difference in summary estimates between strata (Table 6).

### Combined Analysis

Furthermore, we performed a combined analysis of RCTs and observational studies. Statin use was not found to be associated with the risk of lung cancer (RR 0.89, 95% CI 0.77–1.03). The Cochran’s Q test resulted in a *P* < 0.001 (Q =  268.59), and the corresponding quantity I^2^ was 93.3%. However, this particular analysis was dominated by the observational studies (14 studies). These studies accounted for the 81.2% in the random-effects model.

## Discussion

This present meta-analysis included 19 clinical studies (5 RCTs and 14 observational studies), involving a total of 5,009,404 participants and 38,013 lung cancer cases. Overall, both meta-analyses of RCTs and observational studies showed no evidence for an association between statin use and the risk of lung cancer. Our results are in accord with recent meta-analyses on the association between statin use and other site-specific cancers. Likewise, they concluded that statins do not offer any substantial increase or reduction in colorectal, pancreatic, melanoma, or breast cancer risk [47]–[50].

In our subgroup analyses, the results were not substantially affected by study design, RCTs of lipophilic or lipophobic statins, and studies of long-term statin use, which reinforce our confidence in the validity of the conclusion that statin use was not associated with lung cancer risk. Although significant heterogeneity was observed among cohort and case-control studies, summary estimates were essentially unchanged after excluding the studies contributing most to the heterogeneity.

Several meta-analyses have evaluated the association between statins and lung cancer risk [51]–[53]. In the meta-analysis of twenty case-control studies [51], Taylor et al found a significant association between statin usage and any cancer, but when stratified by cancer type, only the association with colon cancer remained. However, the studies were significantly heterogeneous (*P* < 0.01) and these case-control studies were susceptible to various biases. A 2007 meta-analysis included RCTs and observational studies concluded that statin use was not associated with lung cancer risk. And this meta-analysis included 12 observational studies, only 3 were limited to lung cancer [52]. The recent meta-analysis by Kuoppala et al. [53] used hierarchical quality-based methods in evaluation and contained several observational studies not included in previous reports. This meta-analysis showed that statins had no effect on the incidence of lung cancer. However, the effect estimate for lung cancer had wide range (median RR 0.92, range 0.83 to 3.0) and the strength of evidence was weak.

Although we found no association between statin use and lung cancer risk in clinical studies, several preclinical studies indicate that statins may have cancer chemopreventive properties. The mechanistic data suggest that statins’ chemopreventive potential against cancer through their inhibition of the mevalonate pathway [54]. The mevalonate pathway is an important metabolic pathway that provide cell with bioactive molecules which play a key role in multiple cellular processes such as membrane integrity, cell signaling, protein synthesis, and cell cycle progression [55]. Statins’ inhibition of HMG-CoA reductase prevents the conversion of HMG-CoA to mevalonate, and thereby reduce levels of mevalonate and its downstream products, probably resulting in control of tumor initiation, growth, and metastasis [56], [57].

Increasing evidence also suggests that statins might enhance the antitumor activity of various cytokines and chemotherapeutic agents. In a phase 2 study of irinotecan, cisplatin, and simvastatin for untreated extensive-disease small cell **lung cancer** (ED-SCLC), the results indicated that the addition of simvastatin to irinotecan and cisplatin might improve the outcome of heavy smokers with ED-SCLC [58]. And another phase 2 study of gefitinib plus simvastatin versus gefitinib alone showed that simvastatin might improve the efficacy of gefitinib in that subgroup of gefitinib-resistant non-SCLC patients [59]. Because this field is new, only a few clinical trials have been reported so far. Therefore, the combined treatment of tumors with statins and anticancer drugs is an area of research that warrants future study.

Interestingly, the recent study by Nielsen et al [60] suggested that statin use was associated with a substantial decline in cancer-related mortality. They assessed mortality among patients from the entire Danish population who had received a diagnosis of cancer between 1995 and 2007. The study design provided substantial power to evaluate mortality from cancer with limited selection bias. However, the study does have some major limitations that may influence the interpretation of the results [61]. One limitation is that the important information on smoking and other risk factors (such as surgery) are not available. Another limitation is that there is no clear pattern of decreased mortality with increased dose [61]. Therefore, more studies are needed to verify the findings in other populations, taking into account treatment, smoking status, and other risk factors.

The present study has several strengths. First, this latest meta-analysis combined all relevant literature published up to March of 2012. Moreover, 19 clinical studies were included in our meta-analysis, reporting data of 38,013 lung cancer cases. Meta-analysis of studies with large numbers of incident cases provides high statistical power for estimating the relationship between exposure and outcome risk.

Nevertheless, our meta-analysis has several limitations. First, the included studies were different in terms of study design and definitions of drug exposure. However, our findings were stable and robust in the subgroup analyses. Second, a meta-analysis is not able to solve problems with confounding factors that could be inherent in the included studies. Inadequate control for confounders may bias the results in either direction, toward exaggeration or underestimation of risk estimates. In our meta-analysis, each observational study adjusted RR for different confounding factors which might be a source of heterogeneity. Third, heterogeneity may also be introduced because of methodologic and demographic differences among studies. We used appropriate well-motivated inclusion criteria to maximize homogeneity, and performed sensitivity and subgroup analyses to investigate potential sources of heterogeneity. Finally, inherent in any review process of published studies is the possibility of publication bias. Our search was restricted to studies published in indexed journals. In this meta-analysis, we did not search for unpublished studies or for original data. However, we found no evidence of substantial publication bias.

In summary, findings from this meta-analysis indicated that statin use was not associated with the risk of lung cancer.

## Supporting Information

File S1 **PRISMA Checklist for the meta-analysis.**
(DOC)
